# A Differential Evolution Algorithm Based on Nikaido-Isoda Function for Solving Nash Equilibrium in Nonlinear Continuous Games

**DOI:** 10.1371/journal.pone.0161634

**Published:** 2016-09-02

**Authors:** Feng He, Wei Zhang, Guoqiang Zhang

**Affiliations:** 1 College of Management and Economics, Tianjin University, Tianjin, China; 2 Xuchang University, Xuchang, China; East China University of Science and Technology, CHINA

## Abstract

A differential evolution algorithm for solving Nash equilibrium in nonlinear continuous games is presented in this paper, called NIDE (Nikaido-Isoda differential evolution). At each generation, parent and child strategy profiles are compared one by one pairwisely, adapting Nikaido-Isoda function as fitness function. In practice, the NE of nonlinear game model with cubic cost function and quadratic demand function is solved, and this method could also be applied to non-concave payoff functions. Moreover, the NIDE is compared with the existing Nash Domination Evolutionary Multiplayer Optimization (NDEMO), the result showed that NIDE was significantly better than NDEMO with less iterations and shorter running time. These numerical examples suggested that the NIDE method is potentially useful.

## Introduction

Nash equilibrium(NE) is one of the most important concepts in game theory, since it reveals the intrinsic link between the game equilibrium and economic equilibrium. NE is widely used in practical applications, therefore, it is a key issue to find NE. However, infinite strategies and complex payoff functions (such as non-concave, non-differentiable, multi-modal, etc.)make it difficult to solve NE. In fact, the difficulty of solving the NE has greatly limited its application in economics, management and sociology.

Economists mainly studied continuous games, in which there are infinite strategies and the payoff functions are also continuous. As we all know, the purpose of game analysis is to predicate a result of a game, which is NE. At present, the commonly used methods for locating NE of a continuous game are the following.

(1) Analytical method is the most straightforward approach, which sets the partial derivatives of every player to zero[1], thus we can obtain the optimum strategy for one player if the other players do not change their strategies. This is also known as player’s best-response function. Solving all the best-response function can obtain the optimal strategy of each player, that is NE[2,3]. The analytical method is useful for providing insights into the behavior of players, however, it is difficult to apply this method to deal with multi-player or non-differential payoff function [4,5].

(2) Mathematical programming is the most widely used method in detecting NE, which is convenient to add certain constraint to the model, and easy to extend to a big system. The mathematical programming method can be divided into the following categories: sensitivity information based [6]; non-interior point algorithm[7]; nonlinear complementarity approach[8], etc. Furthermore, a variational inequality approach [9] has also been applied in Cournot model. However, the mathematical programming method is heavily dependent on the choice of the initial point. If we set an improper initial point, the solution may only be a local optimal solution.

(3) Matrix game is a method which disperses the strategy space, therefore, each player's strategy space is composed of several typical strategies [10]. In addition, the matrix game is also used in the mixed NE[11].

(4) Evolutionary algorithm is a stochastic optimization method, which is not strictly limited to the objective function and can also handling non-differentiable, nonlinear and other complex payoff functions. Son[12] proposed an improved co-evolutionary algorithm—hybrid co-evolutionary programming to find a global optimal NE, which overcame the local optimal shortcoming and improved the convergence of the co-evolutionary algorithm. There are also other optimization algorithms such as invasive weed optimization[13]; cooperative co-evolutionary invasive weed optimization[14]; PSO[15], etc.

In many economic models, there are some special functions to convert NE into a optimization problem, such as Nikaido-Isoda function[16] and Lyapunov function[17]. Moreover, equilibrium also can be described as solution of polynomial equations [18].

When building a game model, economists generally assume that the demand curves are monotonically decreasing functions, and the cost functions are constant or represented by a quadratic functions. The advantage of this assumption is to guarantee a unique NE, which is convenient to find NE. In real economic activity, however, the nonlinear demand functions and cost functions made the model more consistent with the real market competition, this will inevitably lead to the payoff functions become extremely complex, thus making the NE difficult to be solved.

A differential evolution algorithm(DE) for solving Nash equilibrium in nonlinear continuous games is presented in this paper, called NIDE. In each generation, parent and child strategy profiles are compared one by one pairwisely with Nikaido-Isoda as fitness function. In applied research, the NE of nonlinear game model with cubic cost function and quadratic demand function(even some other non-concave payoff functions) are solved. Compared with the existing Nash Domination Evolutionary Multiplayer Optimization (NDEMO), the results suggested that the NIDE was significantly better than NDEMO with less iterations and shorter runtime for solving the NE problems. Meanwhile, it illustrated that the NIDE has high efficiency and wide applicability.

The organization of this paper is as following. Section 2 explains the basic concepts of game theory and provides a searching algorithm based on DE. Section 3 presents numerical examples and compared with NDEMO, and finally conclusions are drawn afterwards

## Materials and Methods

### Prerequisites

The normal-form game usually consists of three parts:

N = {1,⋯,n} denotes the set of players, n is the number of players;

$S_{i}=\{S_{i1},S_{i2},\cdots ,S_{{im}_{i}}\}$ denotes the strategies set of player i;

u_i_: S → R denotes the payoff function for each player i ∈ N, where S = S_1_ × S_2_ × ⋯ × S_n_ is a Cartesian product and represents all possible situation of the game, and R is set of real number.

Given a particular topology for a set S_i_, there is corresponding direct product topology for the set S = S_1_ × S_2_ × ⋯ × S_n_. For example, if S_i_ is a compact metric space, then S is also a compact metric space. As players’ strategies can be quantified, so the pure strategy of each player S_i_ can be regarded as a bounded and closed convex subset in Euclidean space. If the number of strategies S_i_ for player i is infinite, it is called “Infinite game”.

Definition 1: The infinite game G = {s_1_, ⋯, s_n_; μ_1_, ⋯, μ_n_} is called continuous game, if given a certain topology for a set S, and the payoff function u_i_: S → R is a continuous function for player i ∈ N.

In an N-player game, when player i chooses his pure strategy s_i_ ∈ S_i_ given a pure strategy set of others s_−i_ = (s_1_, ⋯, s_i−1_,s_i+1_ ⋯, s_n_) ∈ s_−i_, he receives a profit of u_i_(s_i_,s_−i_). In order to study the equilibrium of continuous game, we need introduce the concept of NE.

**Definition 2:** In the normal-form game G = {s_1_, ⋯, s_n_; μ_1_, ⋯, μ_n_}, a combined strategy profile $s^{*}=(s_{1}^{*},\cdots ,s_{i}^{*},\cdots ,s_{n}^{*})$ is an NE if

$${\;u}_{i}(s_{i}^{*},s_{-i}^{*})\geq u_{i}(s_{i},s_{-i}^{*}),\forall s_{i}\in S_{i},\forall i\in N,$$ (1)

NE is a point where no player can obtain a higher profit by unilateral movement. That is, a profit-maximizing player will not deviate from NE. In fact, if a strategy profile $s^{'}=(s_{1}^{'},\cdots ,s_{i}^{'},\cdots ,s_{n}^{'})$ is not NE, that means there must exist a player i who can obtain a higher profit by choosing another strategy $s_{i}^{"}$ when others choose $s_{-i}^{'}$, that is $u_{i}(s_{i}^{"},s_{-i}^{'})>u_{i}(s_{i}^{'},s_{-i}^{'})$.

**Definition 3:** In an N-player game, a strategy $s_{i}^{*}$ is called “dominant strategy” for player i, if

$${\;u}_{i}(s_{i}^{*},s_{-i})>u_{i}(s_{i}^{'},s_{-i}),\forall s_{-i}\in S_{-i},\forall s^{'}\;\in S_{i},\;s^{'}\;\neq s_{i}^{*}$$ (2)

Accordingly, the strategy $s_{i}^{'}$ is called dominated strategy. From the definition 2, if $s_{i}^{*}\in S$ is a dominant strategy for player i, the outcome resulting from the strategy $\;s_{i}^{*}$ is better than the outcome resulting from any strategy $s^{'}\in S_{i}(s^{'}\neq s_{i}^{*})$, regardless of what the other players are doing.

Sometimes, a strategy to be a dominant strategy is only relative to the specific strategy, thus we have the following definition.

**Definition 4:** In a game, a player may have two strategies $s_{i}^{'}$ and $s_{i}^{"}$, then strategy $s_{i}^{"}$ is said to relatively dominant strategy $s_{i}^{'}$, than is

$$u_{i}(s_{i}^{'},s_{-i})<u_{i}(s_{i}^{"},s_{-i}),\forall s_{-i}$$ (3)

It means that there may exist another better strategy than $s_{i}^{"}$.

Next, we will introduce the concept of Nikaido-Isoda function[19], which plays an important role in the study of NE.

**Definition 5:** Let u_i_ be the payoff function of player i, then the Nikaido-Isoda function ψ: (S_1_ × S_2_ × ⋯ × S_n_) × (S_1_ × S_2_ × ⋯ × S_n_) → R is

$$\psi (x,y)=\sum_{i=1}^{n}\left[{\;u}_{i}(y_{i}|x)-{\;u}_{i}(x)\right].$$ (4)

Each term of the series in the Nikaido-Isoda function represents the change of one player’s gains from strategy x_i_ to y_i_, while the strategies of the others are held fixed. Nikaido-Isoda function represents the sum of the change in gains or losses of all players.

Based on the definition 4 and definition 5, given two strategy profiles

$$\{x,y\}\in S,(x\equiv ({\;x}_{1},\cdots ,{\;x}_{n}),y\equiv ({\;y}_{1},\cdots ,{\;y}_{n})),\;{\;y}_{i}\neq {\;x}_{i},$$

if u_i_(y_i_|x) > u_i_(x) always hold for each player i ∈ N, we say the strategy profile y is dominant to the strategy profile x. at this point, we have the following proposition hold.

**Proposition 1:** If the strategy profile y is dominant to the strategy profile x, then

ψ(y,x)<ψ(x,y). (5)

Prove: If the strategy profile y is dominant to the strategy profile x, then

$$u_{i}({\;x}_{i},s_{-i})<u_{i}({\;y}_{i},s_{-i}),\forall s_{-i}$$

substitute it into Eq (4), we have

$$\psi (x,y)=\sum_{i=1}^{n}[{\;u}_{i}(y_{i}|x)-{\;u}_{i}(x)]>0$$

Similarly,

$$\psi (y,x)=\sum_{i=1}^{n}\left[{\;u}_{i}(x_{i}|y)-{\;u}_{i}(y)\right]<0$$

Therefore, ψ(y,x) < *ψ*(x,y) holds.

If x* is NE, for any y ∈ S, and y ≠ x*, then ψ(x*,y) < *ψ*(y,x*). Therefore the Nikaido-Isoda function plays the role of a “merit function” measuring the proximity of a strategy to NE.

### NIDE algorithm

In 1995, Storn and Price[20–22] proposed a advanced evolutionary algorithm, called differential evolutionary(DE), which was a relatively new stochastic optimization method, and has been shown to be a simple and effective algorithm for the problem of continuous global optimization[21]. The DE requires few control variables, is robust, easy to use and lends itself very well to parallel computation.

There are three important variables in DE: Mutation Factor F, Probability of Crossover CR and Population size NP. Mutation Factor F is positive real number which controls the amplification of the differential variation. Experience has shown that, it is best to choose F between 0.4 and 1.0. In addition, a crossover operation is performed, using the parameter CR, the value of CR is smaller, the robustness of DE will be better. Generally, CR ∈ [0,1]. If the population size NP is larger, the search ability of DE will be intensified, and the calculated amount of DE will be increased. According to literature [21], NP usually to be chosen between 5n–10n, where n is the number of variables.

Based on Proposition 1, by redefinition of selection criteria used in evolutionary methods with the Nikaido-Isoda function, we propose a differential evolution(NIDE) to find the NE. At each generation, parent and child strategy profiles are compared one by one pairwise, using Nikaido-Isoda function to play for the part of fitness function. The whole process can be summarized into the following steps(Algorithm1).

**Algorithm1:** A differential evolution based on Nikaido-Isoda function(NIDE)

1: Input: the maximum number of iterations Max_it_, the population size NP, Mutation Factor F, Probability of Crossover CR, the convergence tolerance ε

2: it ← 1

3: Randomly initialize populations of NP parent strategy profiles P

4: Evaluate fitness to players with P

5: **while** it < Max_it_ **do**

6: **for** i = 1 to NP **do**

7: $x\leftarrow P_{i}^{it}$

8: Use Algorithm 2 to create child strategy profiles y

9: **if** ψ(y,x) < *ψ*(x,y) **then**

10: $P_{i}^{it+1}\leftarrow y$

11: **end if**

12: **end for**

13: Randomly choose a chromosome s from P

14: Compute Euclidean norm between s and every member in P

15: **if** maximum of norm ≤ ε **then**

16: Terminate

17: **else**

18: it ← it + 1

19: **end if**

20: **end while**

21: Output: Nash Solutions

Child strategy profiles y are created by applying the DE operators via the stochastic combination of randomly chosen parents as discussed in Storn(2003) [23]. Through tested on five different forms of DE operators(DE/rand/1/bin, DE/best/1/bin, DE/rand/2/bin, DE/best/2/bin, DE/rand–to–best/1/bin), we found the operator “DE/rand/1/bin” possessed faster convergence. In order to show the performance of NIDE, we use the same algorithm in literature Koh(2012)[24] to create child strategy profiles, just describe by the following(Algorithm 2).

**Algorithm 2:** Creating a child vector via DE

1: Input: Current Population P

2: Input: Mutation Factor F, Probability of Crossover CR

3: Input: Lower Bounds LB_d_ and Upper Bounds UB_d_ in each dimension d

4: Randomly choose 3 integers: r_1_, r_2_, r_3_ between 1 and NP

5: r_1_ ≠ j, r_2_ ≠ j and r_3_ ≠ r_2_ ≠ r_1_ ≠ j

6: x ← P_j_

7: $a\leftarrow P_{r_{1}}$

8: $b\leftarrow P_{r_{2}}$

9: $c\leftarrow P_{r_{3}}$

10: Mutation: Produce a mutant vector z via a stochastic combination of donor vectors

11: z ← a + F(b−c)

12: **for** d = 1 to D **do**

13: Crossover

14: $y_{d}\leftarrow \left\{ \begin{array}{l} z_{d}\;if\;rand(0,1)<CR\vee d=intr(1,D)\\ \;x_{d}\;otherwise \end{array}\right.$

15: Enforce Bound Constraints

16: $y_{d}\leftarrow \left\{ \begin{array}{l} (x_{d}+{LB}_{d})/2\;if\;y_{d}<{LB}_{d}\\ (x_{d}+{UB}_{d})/2\;if\;y_{d}>{LB}_{d}\\ \;y_{d}\;otherwise\; \end{array}\right.$

17: **end for**

18: Output: child vector y

In order to check convergence to the NE, randomly select a individual s and compute the Euclidean norm between s and every member of P(lines 13 to 14 of Algorithm1). If the maximum distance is less than ε, the population is judged to have converged to a NE and the algorithm can terminated. Otherwise the process is repeated.

## Results and Discussion

In order to test the efficiency of the NIDE, we make a comparison between NIDE and NDEMO[24]. Table 1 gives the parameters used for the numerical experiments just the same in Koh(2012)[24]. All numerical experiments were conducted using Matlab R2013a running on a 32 bit Windows 7 machine with 2 GB of RAM.

**Table 1:** Parameters used in the NIDE for all numerical experiments.

Parameter name	Parameter values
Maximum number of iterations	400
Population size	50
Probability of crossover	0.35
Mutation factor	0.45
Termination tolerance	0.0001

### Oligopolistic game model with cubic cost function

When building a game model, Economists generally assume that the demand curves are monotonically decreasing functions, and the cost functions are constant or represented by a quadratic functions. The advantage of this assumption is there exists a unique NE, and it is convenient to finding the NE. In real economic activity, however, the nonlinear demand functions and cost functions make the model more consistent with the real market competition, this will inevitably lead to the payoff functions become extremely complex, thus making the NE difficult to be solved.

In microeconomics, however, the total cost function is always non-negative in its definitional domain and decreases to zero on the left side of the inflection point, then it gradually increases while on the right side of the inflection point[25]. The literature [25] pointed out the total cost curve is the analogy of cubic function, so we employ

$$f(x)=a+bx+{cx}^{2}+{dx}^{3}(d\neq 0)$$ (6)

There are four forms of cubic function. d > 0 and Δ ≤ 0; d < *0* and Δ ≤ 0; if d < *0* and Δ > 0; d < 0 and Δ > 0 and Δ = 4c^2^ − 12bd.

The total cost function is a cubic function as a > 0, d > 0, Δ ≤ 0, and the inflection point ($-\frac{c}{3d}$, $\;f\left(-\frac{c}{3d}\right)$) falls in the first quadrant, that is,

{a>0d>0∆=4c2−12bd≤0−c3d>0f(−c3d)=2c3−9bcd+27ad2>0 (7)

#### Case 1: Example 1 with two players

Here we first consider the situation with two players i, i = 1,2. Suppose that the output of player i is q_i_, each player maximizes individual profits from the sale of the homogeneous good. The total outputs are

$$Q=q_{1}+q_{2},$$

and the inverse demand function[26] is

$$P=P(Q)=m-nQ^{2}.$$

The total cost curve is

$$C_{i}(q_{i})=a_{i}+b_{i}q_{i}+c_{i}q_{i}^{2}+d_{i}q_{i}^{3},i=1,2$$

Hence the profit function of player i is given by

$${\;\pi }_{i}(q_{1},q_{2})=q_{i}[m-nQ^{2}]-C_{i}(q_{i}),$$ (8)

We calculate the first order of the profit, i.e.

$$\left\{ \begin{array}{c} \frac{\partial \pi_{1}}{\partial q_{1}}=m-nQ^{2}-2nq_{1}Q-b_{1}-2c_{1}q_{1}-3d_{1}q_{1}^{2}=0\\ \frac{\partial \pi_{2}}{\partial q_{2}}=m-nQ^{2}-2nq_{2}Q-b_{2}-2c_{2}q_{2}-3d_{2}q_{2}^{2}=0 \end{array}\right.$$ (9)

Based on Eq (9), we can find out the player’s response function,

$$\left\{ \begin{array}{c} q_{1}^{*}=\frac{1}{3n+3d_{1}}(-2nq_{2}-c_{1}+\sqrt{M})\\ q_{2}^{*}=\frac{1}{3n+3d_{2}}(-2nq_{1}-c_{2}+\sqrt{N}) \end{array}\right.$$ (10)

Where

$$M=(n^{2}-3nd_{1})q_{2}^{2}+4nc_{1}q_{2}+c_{1}^{2}+3(mn-nb_{1}+md_{1}-b_{1}d_{1})$$

$$N=(n^{2}-3nd_{2})q_{1}^{2}+4nc_{2}q_{1}+c_{2}^{2}+3(mn-nb_{2}+md_{2}-b_{2}d_{2}).$$

It is difficult to obtain the analytical solutions in Eq (10), so we assign a value to each parameter, then use NIDE to detect the NE.

Set

m=5,n=1,

$$a_{1}=0.01,b_{1}=0.4,c_{1}=-0.03,d_{1}=0.005,$$

$$a_{2}=0.01,b_{2}=0.35,c_{2}=-0.025,d_{2}=0.006,$$

where a_i_, b_i_, c_i_, d_i_ submit to the conditions (7).

**Fig 1:**
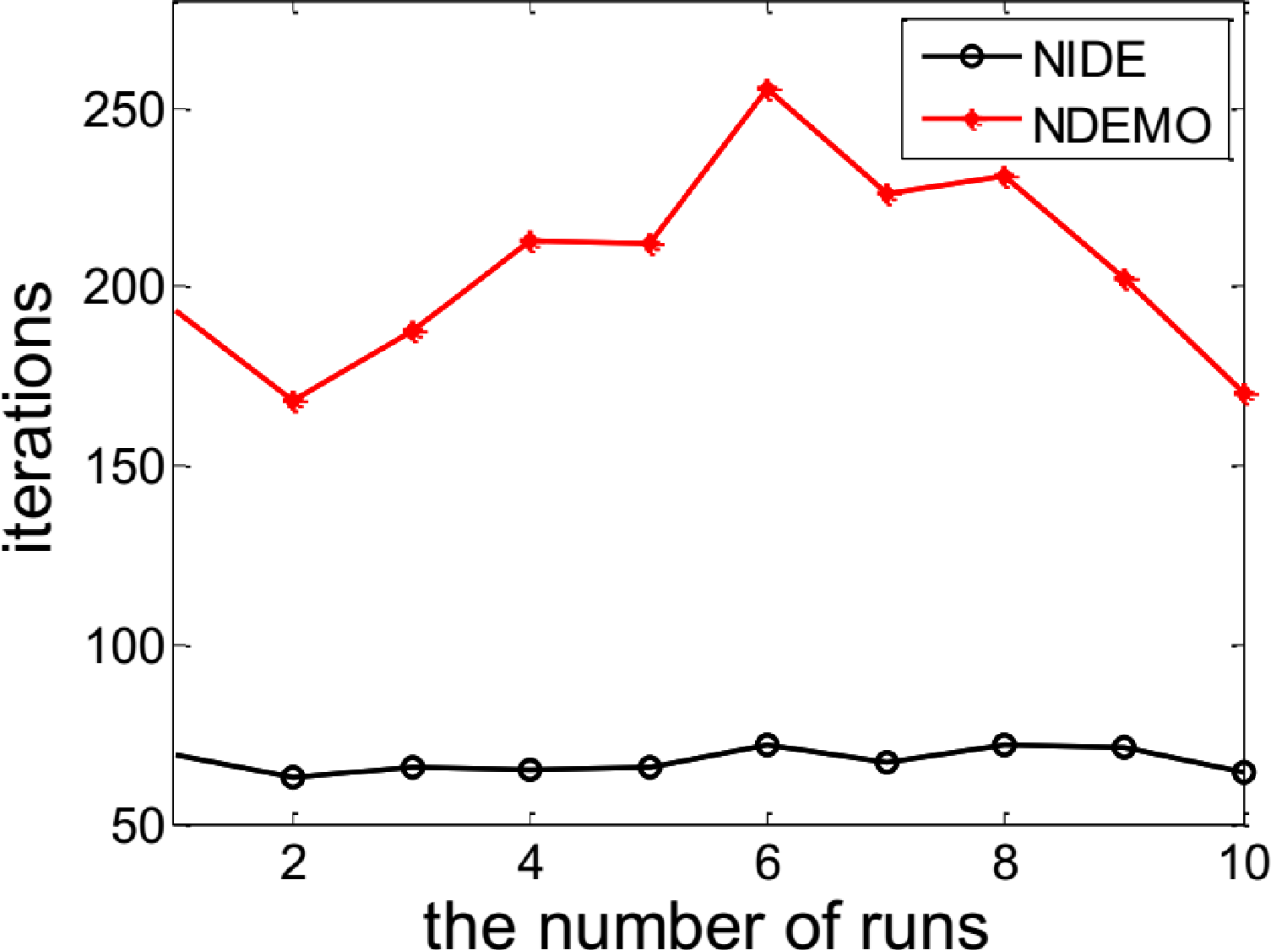
Comparison of iterations between NIDE and NDEMO of Case 1 with two players.

**Fig 2:**
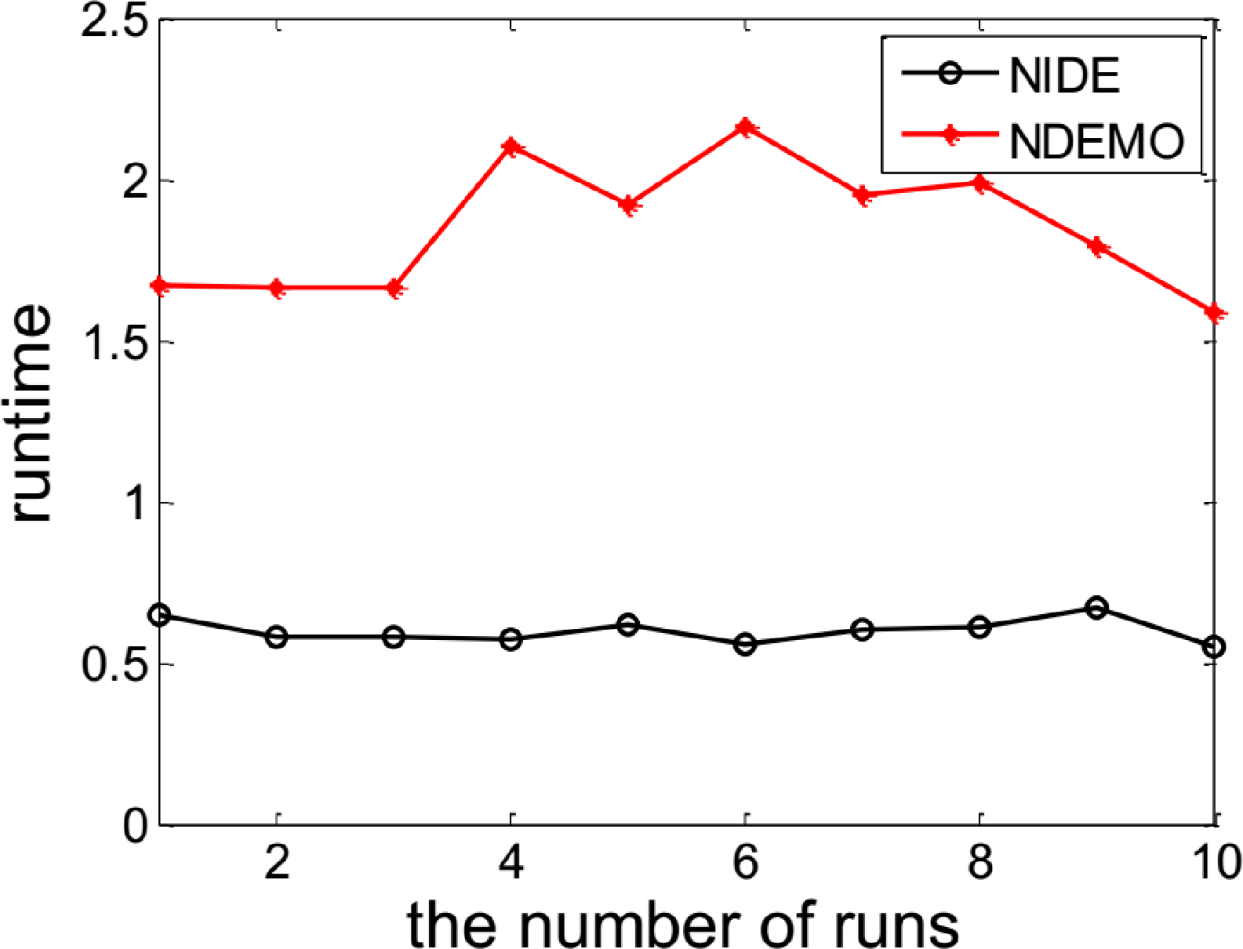
Comparison of runtime of between NIDE and NDEMO Case 1 with two players.

The NIDE identified the NE(0.7563, 0.7697) successfully. In order to compare the iterations and runtime, the NIDE and NDEMO were both runned 10 times, the results were showed in (Fig 1 and Fig 2). The compare results produced by the NIDE and NDEMO were given in Table 2.

**Table 2:** Comparison of the results of NIDE with NDEMO.

Algorithm	NIDE	NDEMO
The average number of iterations	68	206
The average number of runtime(s)	0.60345	1.85430
NE	0.7563, 0.7697	

#### Case 2: Example 1 with three players

For a more complex case, we consider the three player model. We use the same forms of cost and demand function as in Case 1. Three players maximize their profit by adjusting their strategic variable **q**_**1**_, **q**_**2**_ and **q**_**3**_. The profit functions of each player are analogous to those in (8).

$$Set\;m=5,\;n=1;\;a_{1}=0.01,\;b_{1}=0.4,\;c_{1}=\text{-}0.03,\;d_{1}=0.005;\;a_{2}=0.01,\;b_{2}=0.35,\;c_{2}=\text{-}0.025,\;d_{2}=0.006;\;a_{3}=0.01,\;b_{3}=0.3,\;c_{3}=\text{-}0.02,\;d_{3}=0.007.$$

where a_i_, b_i_, c_i_, d_i_ submit to the conditions (7).

**Fig 3:**
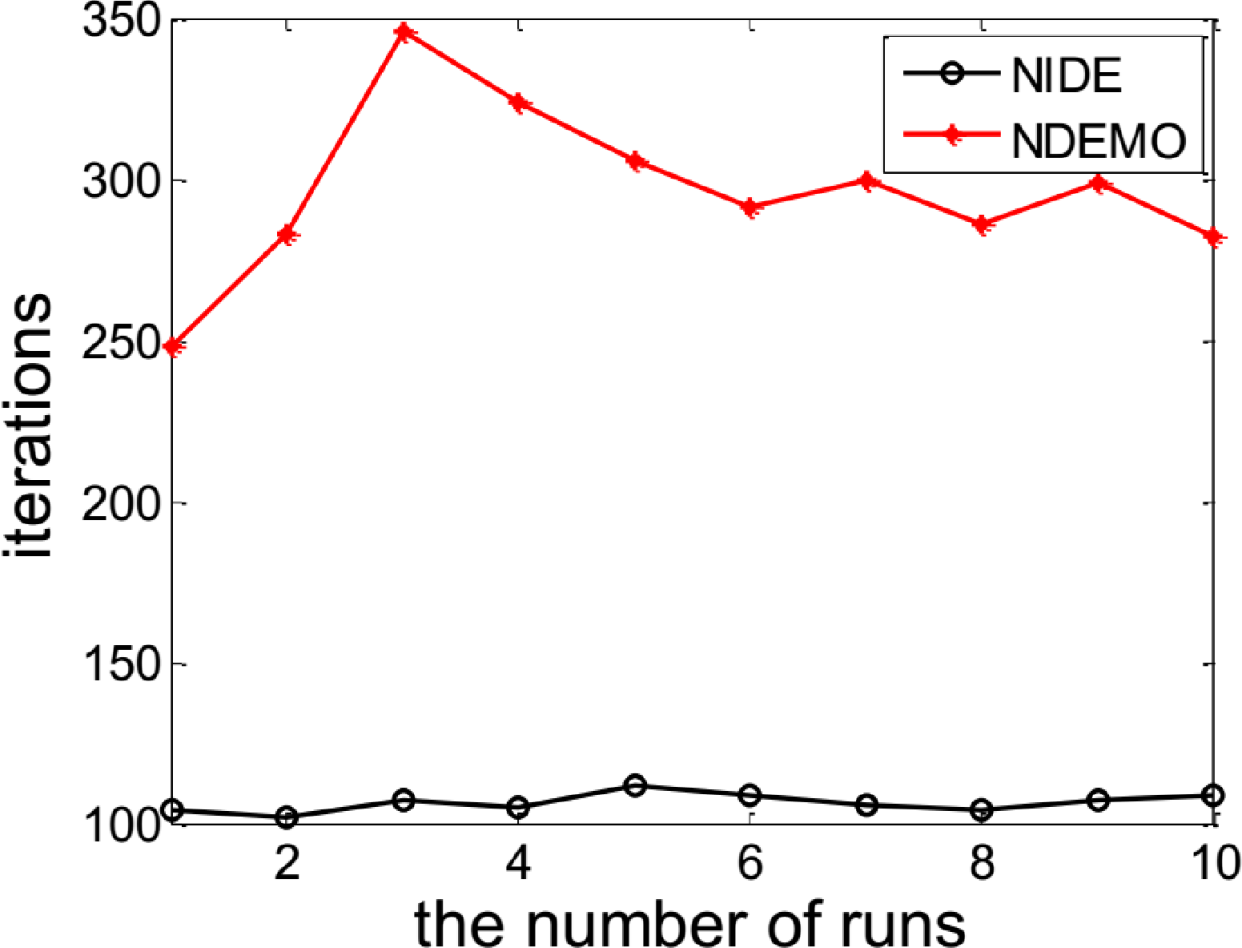
Comparison of iterations between NIDE and NDEMO of Case 2 with two players.

**Fig 4:**
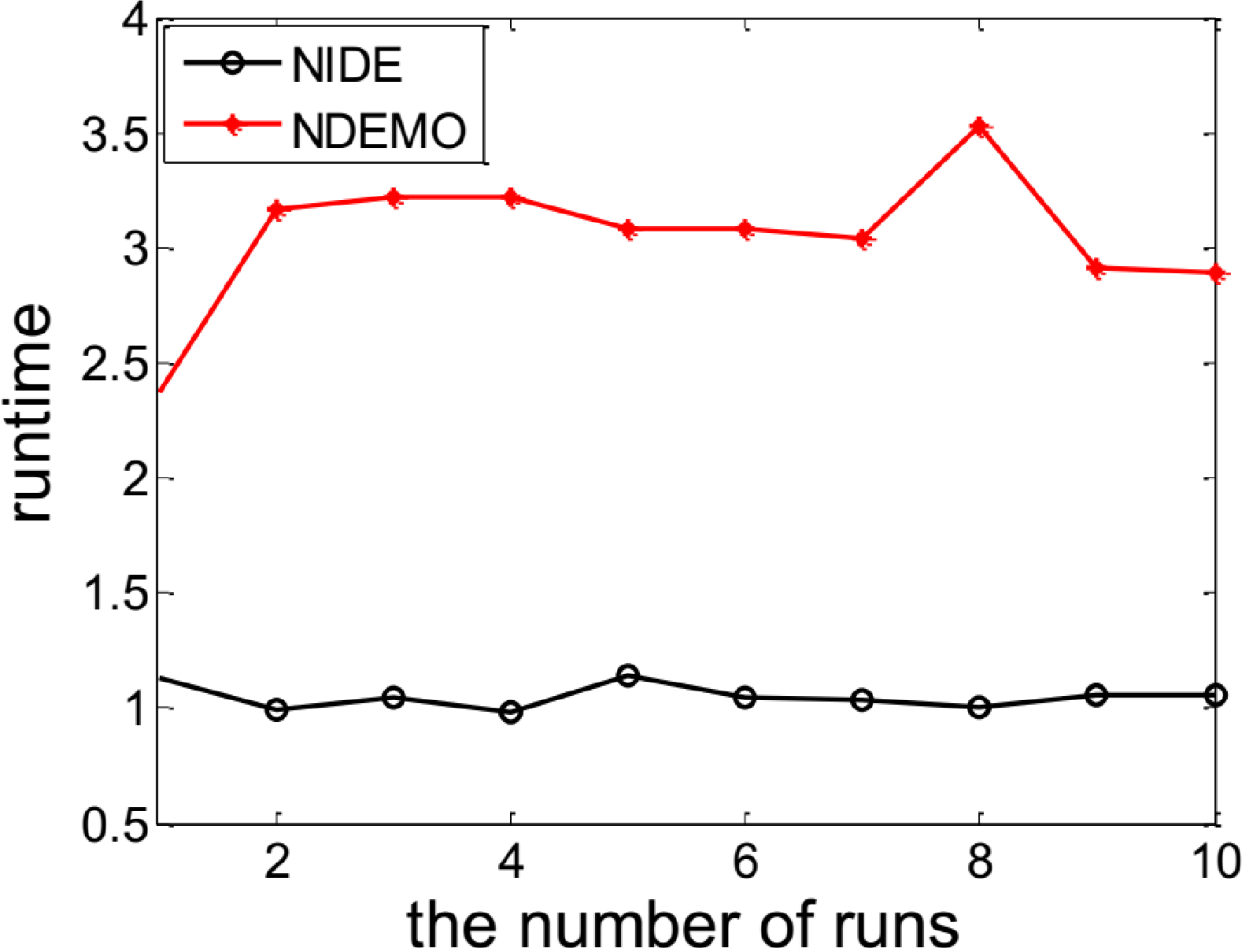
Comparison of runtime of between NIDE and NDEMO of Case 2 with two players.

The NIDE could successfully identify the NE(0.5450, 0.5581, 0.5712)(Fig 3 and Fig 4)as well as Table 3 also show the comparative results of iterations and runtime.

**Table 3:** Comparison of the results of NIDE with NDEMO.

Algorithm	NIDE	NDEMO
The average number of iterations	107	297
The average number of runtime(s)	1.05629	3.05636
NE	0.5450, 0.5581, 0.5712	

Next, in order to show the high efficiency and wide applicability of NIDE, we introduce two more complex models.

### A game model with local NE

This example is taken from [12]. Suppose that there are two players in a market, and the non-concave profit functions for players are defined by

π1(x1,x2)=21+x1sin⁡(pi×x1)+x1x2sin⁡(pi×x2)π2(x1,x2)=21+x2sin⁡(pi×x2)+x1x2sin⁡(pi×x1) (11)

where pi = 3.1415, and the strategic choice of the two players between 0 and 7.5.

We calculate the first order of the profit, i.e.

$$\left\{ \begin{array}{l} \frac{\partial \pi_{1}}{\partial x_{1}}=\sin\left(pi\times x_{1}\right)+pi\times x_{1}cos(pi\times x_{1})+x_{2}\sin(pi\times x_{2})=0\\ \frac{\partial \pi_{2}}{\partial x_{2}}=\sin\left(pi\times x_{2}\right)+pi\times x_{2}cos(pi\times x_{2})+x_{1}\sin(pi\times x_{1})=0 \end{array}\right.$$ (12)

**Fig 5:**
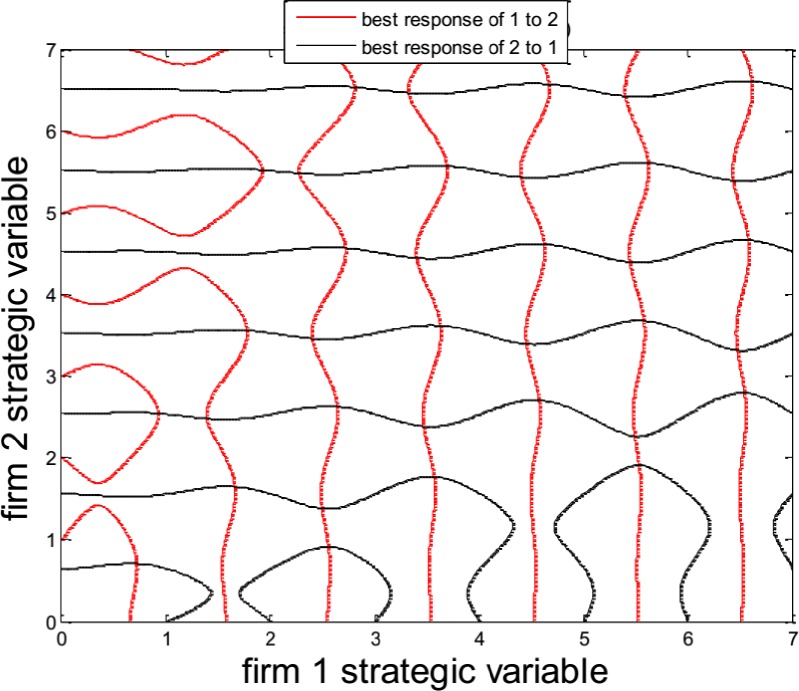
NE in a game model with local NE.

Through (Fig 5), we know that the Eq (12) have many solutions(the points of intersection of Fig 5) in the domain [0, 7.5], and it is difficult to differentiate real NE and“local NE traps” use the conventional NE search algorithms.

**Fig 6:**
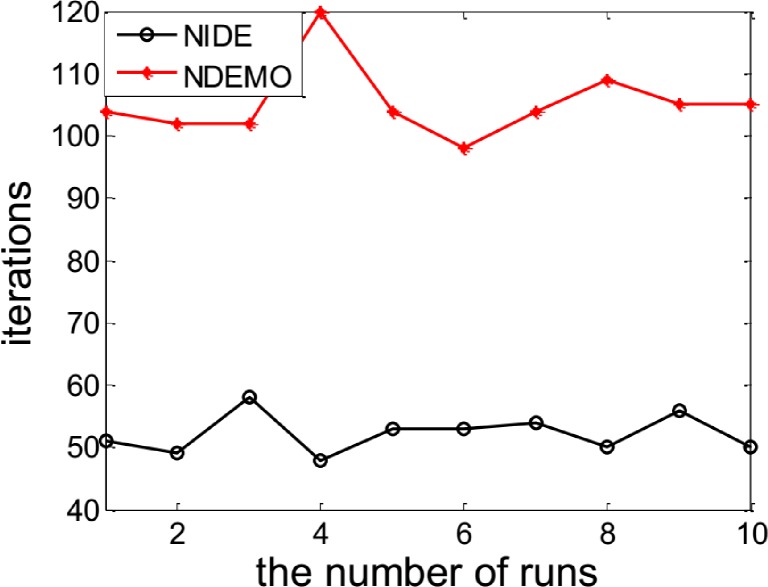
Comparison of iterations between NIDE and NDEMO of local NE game.

**Fig 7:**
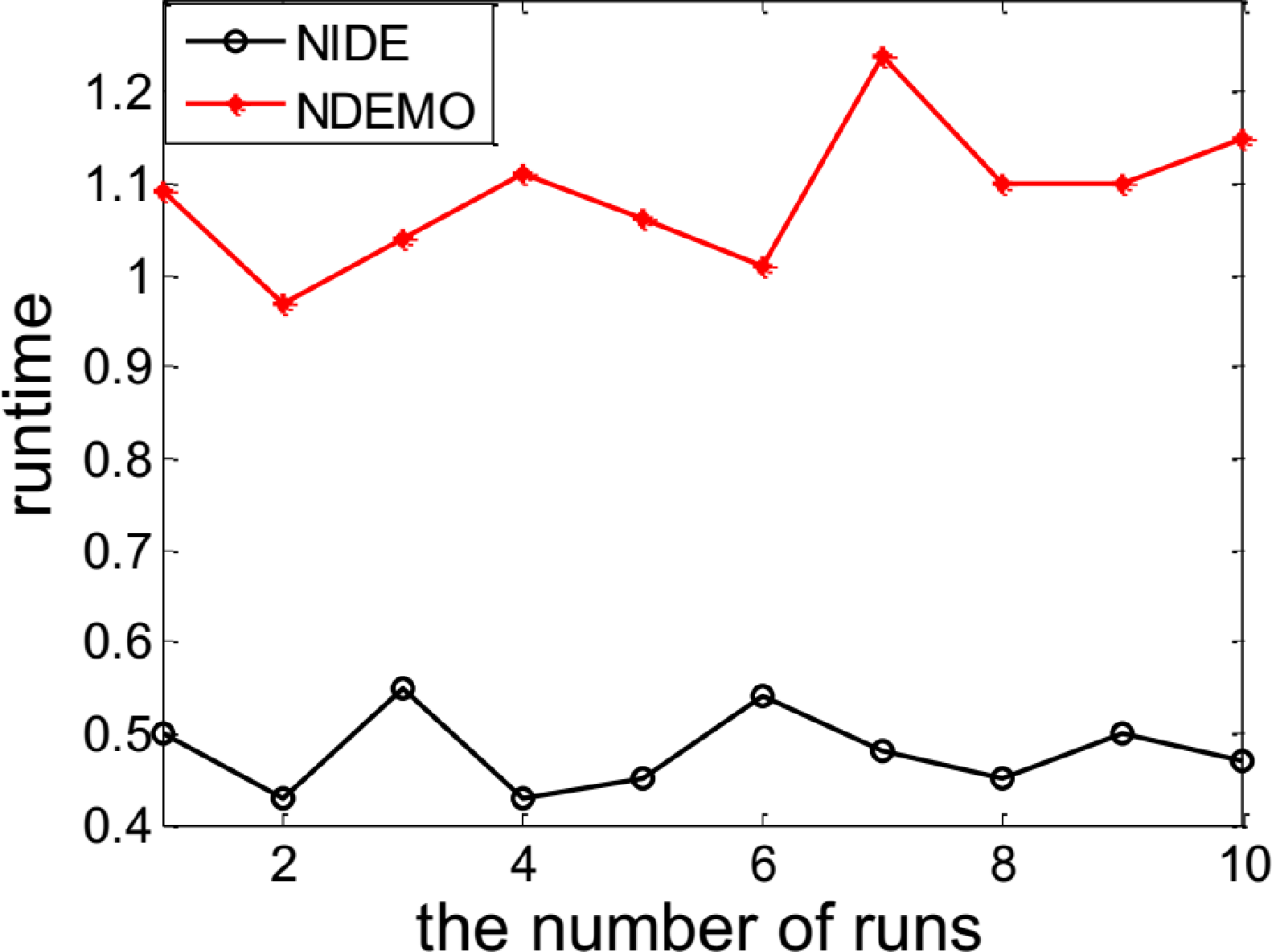
Comparison of runtime of between NIDE and NDEMOof local NE game.

The NIDE overcomes the local optima and detects the NE which agrees with previous results. The comparative results of iterations and runtime are shown in (Fig 6 and Fig 7) and Table 4.

**Table 4:** Comparison of the results of NIDE with NDEMO.

Algorithm	NIDE	NDEMO
The average number of iterations	52	105
The average number of runtime(s)	0.48559	1.09200
NE	6.6118	6.6118

### A game model with 5 players

This example is taken from Koh(2012)[24]. In this example, there are 5 firms, each firm maximizes individual profits from tae sale of the homogeneous good, and inverse demand function is

$$P(q_{1},q_{2},q_{3},q_{4},q_{5})=5000^{1/1.1}{\left(\sum_{i=1}^{5}q_{i}\right)}^{-(1/1.1)},$$

the total cost curve is

$$C_{i}(q_{i})=\omega_{i}q_{i}+(\frac{\theta_{i}}{\theta_{i}+1})\lambda_{i}^{-1/\theta_{i}}q_{i}^{(\theta_{i}+1)/\theta_{i}}.$$

Then, the non-concave profit functions for player i ∈ {1,2,3,4,5} is given by

$$\begin{array}{l} U_{i}(q_{1},q_{2},q_{3},q_{4},q_{5})=P(q_{1},q_{2},q_{3},q_{4},q_{5})q_{i}-C_{i}(q_{i})\\ =(5000^{1/1.1}{\left(\sum_{i=1}^{5}q_{i}\right)}^{-(1/1.1)})q_{i}-\omega_{i}q_{i}-(\frac{\theta_{i}}{\theta_{i}+1})\lambda_{i}^{-1/\theta_{i}}q_{i}^{(\theta_{i}+1)/\theta_{i}} \end{array}$$ (13)

The player dependent parameters shown in Table 5.

**Table 5:** The parameter specification for players.

Firm i	ω_i_	λ_i_	θ_i_
1	10	5	1.2
2	8	5	1.1
3	6	5	1.0
4	4	5	0.9
5	2	5	0.8

(Fig 8 and Fig 9) and Table 6 reports results obtained from applying NIDE and NDEMO to this game.

**Fig 8:**
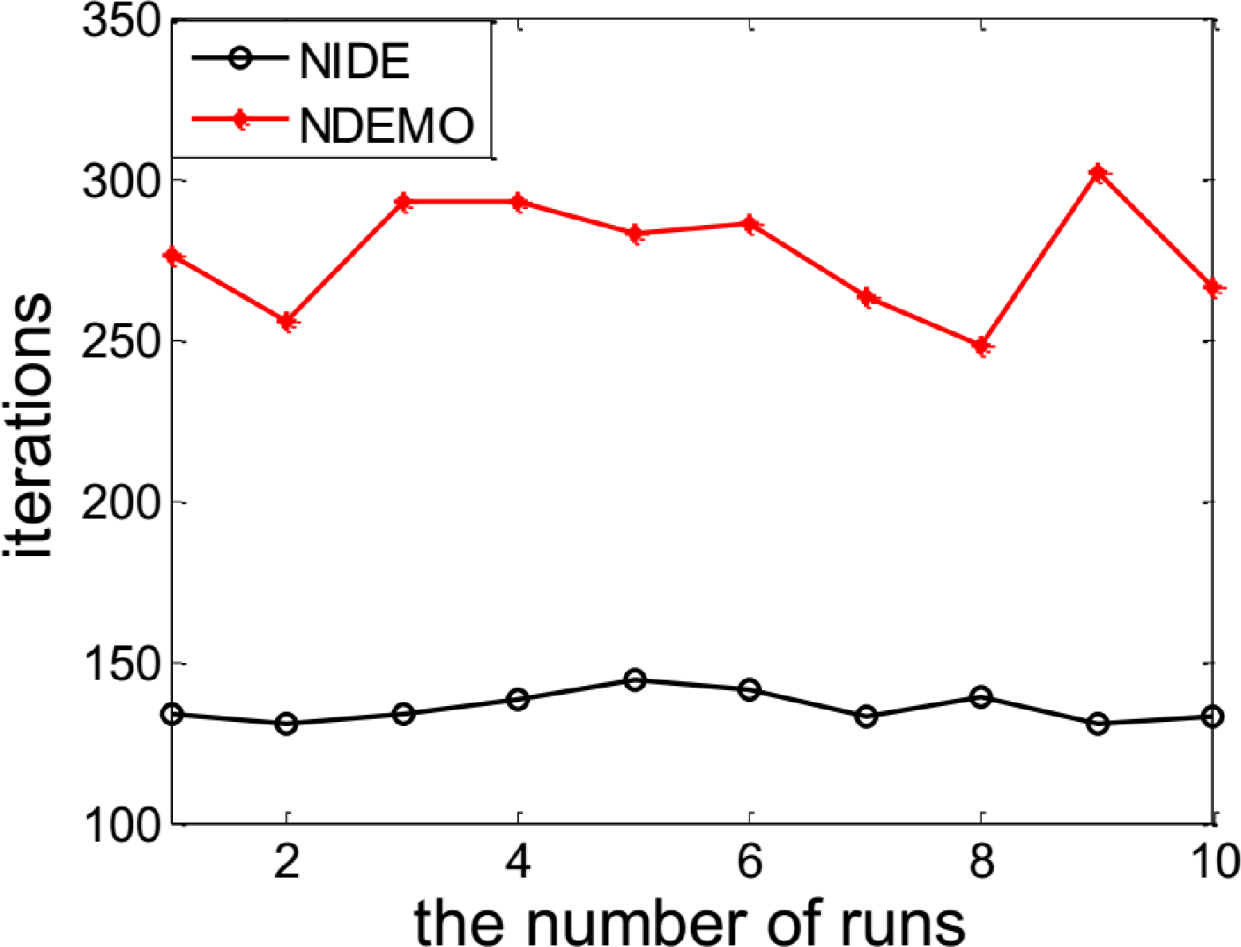
Comparison of iterations between NIDE and NDEMO of a game model with 5 players.

**Fig 9:**
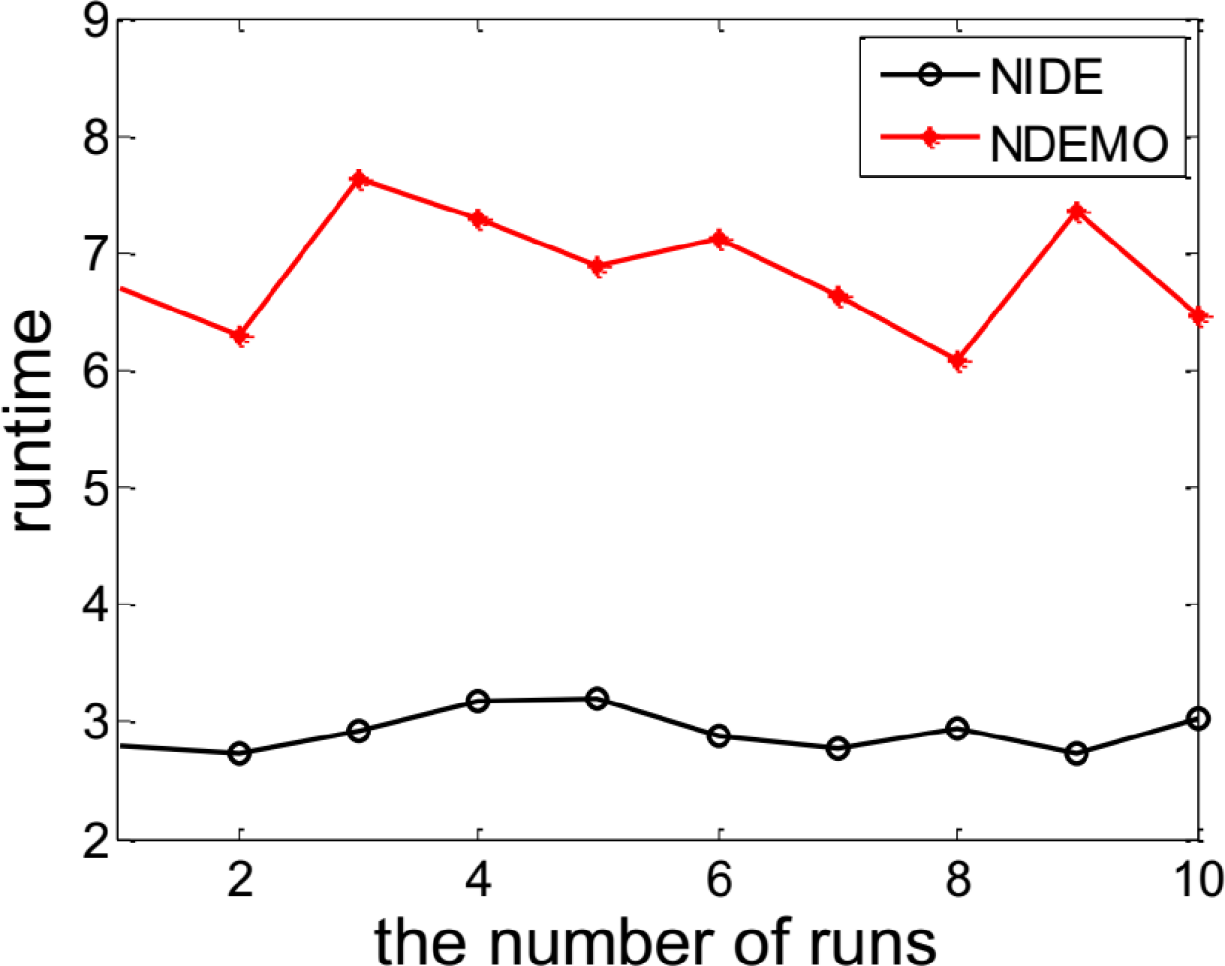
Comparison of runtime of between NIDE and NDEMO of a game model with 5 players.

**Table 6:** Comparison of the results of NIDE with NDEMO.

Algorithm	NIDE	NDEMO
The average number of iterations	136	277
The average number of runtime(s)	2.92571	6.85258
NE	36.9325, 41.8182, 43.7066, 42.6592, 39.1789	

#### Electricity market with two players

Electricity market is a classical linear Cournot model[12]. For sample, we assume there are only two players, and they submit bids to generate quantities s1 and s2, respectively.

In order to analyze the strategy of each player, we define each player’s profit by

π1(s1,s2)=λs1−C1(s1)π2(s1,s2)=λs2−C2(s2) (14)

where λ = [2750-4(s_1_+s_2_)]/75, cost functions C1 = 0.01s_1_^2^+10 s1, and C2 = 0.01s_2_^2^+10 s2.

**Fig 10:**
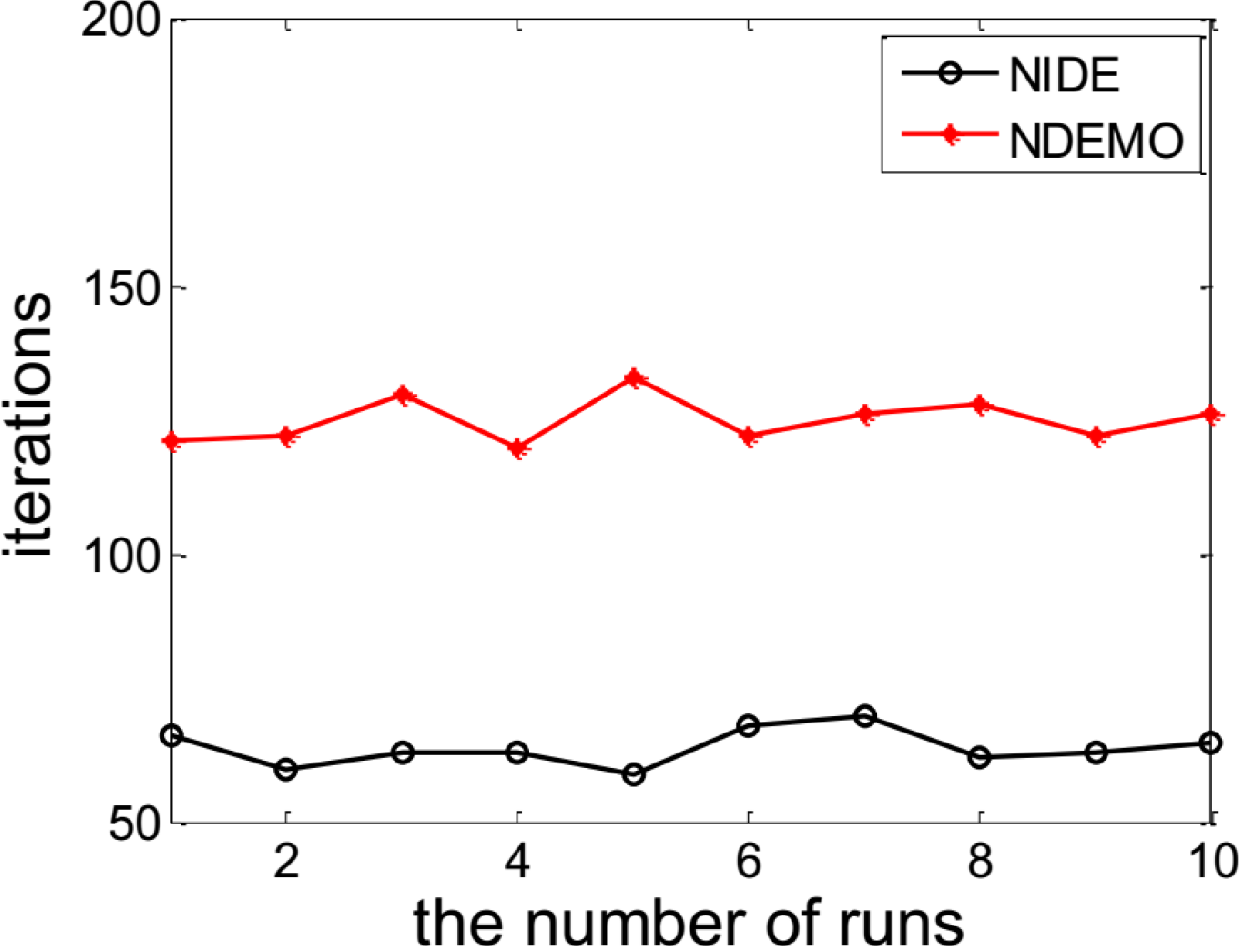
Comparison of iterations between NIDE and NDEMO of Electricity market with two players.

**Fig 11:**
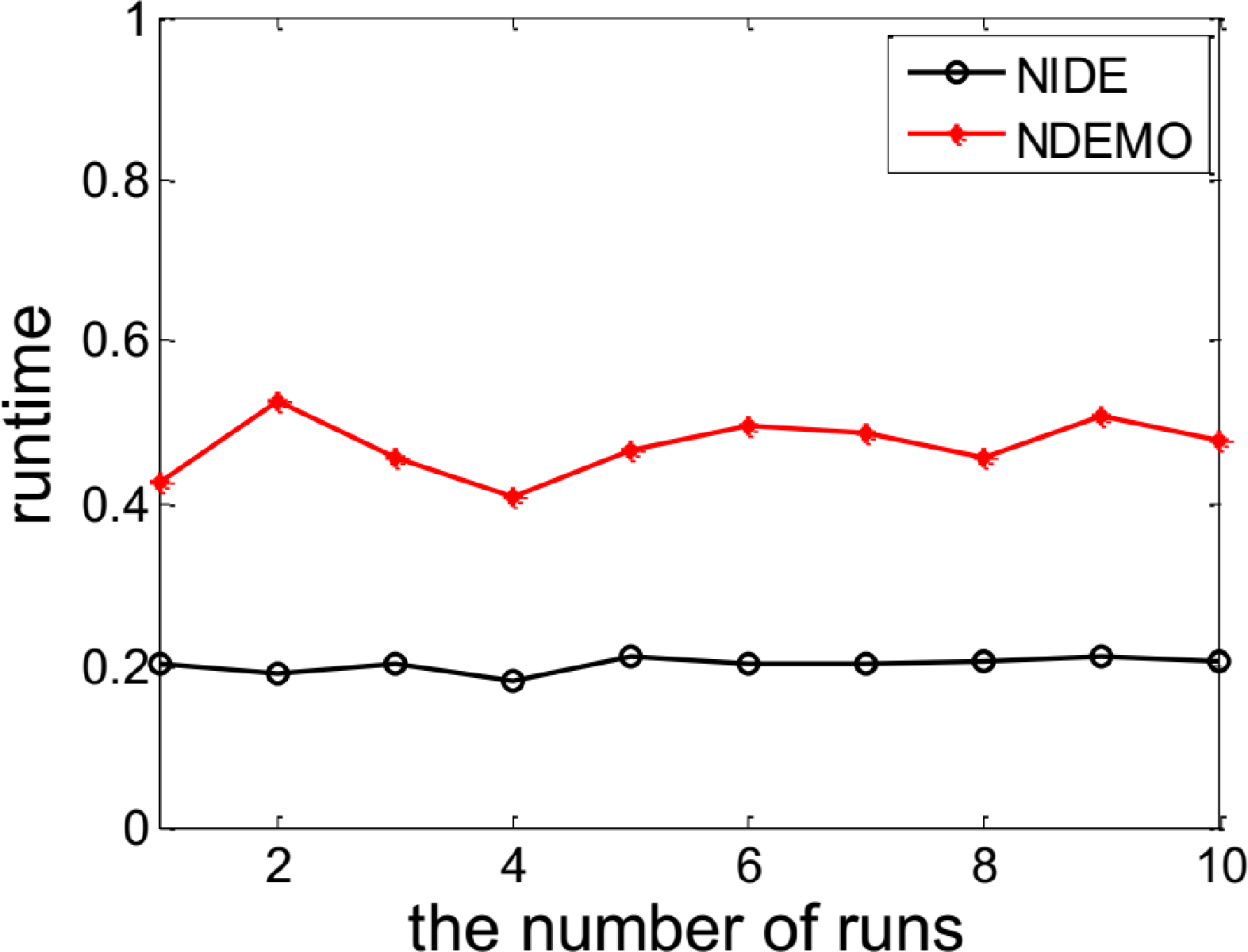
Comparison of runtime of between NIDE and NDEMO of Electricity market with two players.

Fig 10 and Fig 11 and Table 7 reports results obtained from applying NIDE and NDEMO to this game.

**Table 7:** Comparison of the results of NIDE with NDEMO.

Algorithm	NIDE	NDEMO
The average number of iterations	66	122
The average number of runtime(s)	0.20571	0.45258
NE	148.148, 148.148	

From Table 7, NIDE’s average running time is 0.2s with only 66 generations of evolution, while NDEMO took 0.5s with 122 generations. It proved the efficiency of NIDE algorithm is still superior to NDEMO in classical liner demand function model.

## Conclusions

In this paper, we proposed an evolutionary algorithm for solving NE in nonlinear continuous games. The algorithms of NIDE enabled us to handle the NE problems of nonlinear game model with the cubic cost function and the quadratic demand function, and even other non-concave payoff functions.

In order to assess the performance of NIDE, we compared the NIDE with the existing differential evolution algorithm(NDEMO), comparison of the results showed that the NIDE was significantly better than NDEMO with less iterations and runtime for solving the NE problems. It is more efficient in locating NE in practice and will not result in local optimal.

Meanwhile, through these numerical examples, suggesting that the NIDE could be a potentially useful method.
